# Route, early or energy? … Protein improves protein balance in critically ill patients

**DOI:** 10.1186/s13054-018-2015-z

**Published:** 2018-04-14

**Authors:** Peter J. M. Weijs

**Affiliations:** 1 Department of Intensive Care Medicine, VU University Medical Center, Amsterdam, The Netherlands; 2 Department of Nutrition and Dietetics, Internal Medicine, VU University Medical Center Amsterdam, De Boelelaan 1117, 1081 HV Amsterdam, The Netherlands; 3 Department of Nutrition and Dietetics, Faculty of Sports and Nutrition, Amsterdam University of Applied Science, Amsterdam, The Netherlands; 4 Amsterdam Public Health research institute, VU University Medical Center, Amsterdam, The Netherlands

**Keywords:** Protein, Energy, Muscle, Protein balance, High protein feeding, Hypocaloric feeding

## ᅟ

The critical care community still has mixed feelings when considering the optimal nutrition of intensive care unit (ICU) patients, which is understandable as randomized controlled trials have not been very helpful in improving clinical practice. There have been no randomized controlled trials (RCTs) to contribute to the discussion, especially concerning the role of enterally fed protein in optimal critical care.

Recent studies on the route of feeding have shown that enteral nutrition (EN) is not necessarily superior to parenteral nutrition (PN) [1, 2]. There appears to be a strong consensus, with backup from a meta-analysis, on the preferential use of EN over PN [3]. The infection rate was especially used as an argument; however, this is not substantiated in recent trials [1, 2]. We have to consider how applicable this current knowledge is to all ICU patients.

Early EN is still the preferred way of feeding [3]. Starting feeding early may improve the outcome of ICU patients. RCTs have all investigated (supplemental parenteral) energy delivery [4]. Only two trials have ‘considered’ protein: the PERMIT trial [5] (protein supplemented, equal level) and EAT-ICU trial [6] (protein supplemented, higher level). Early energy delivery should be applied cautiously since it appears to be related to worse outcome in ICU patients [7–9]. Therefore, and from the perspective of clinical practice, the Swiss Supplemental PN (SPN) trial appears to provide the most logical design [10]—start with early EN and evaluate on day 3 what the level of energy delivery is; when delivery levels are low (< 60%) start supplementation PN. In clinical practice in our ICU the enteral feeding levels are high enough to avoid PN supplementation, which therefore restricts the specific indication to use PN.

The focus of this research has been caloric delivery. There are more than enough observational data to support that higher protein delivery is associated with improved outcome in ICU patients [7–9]. These observational studies clearly show the benefit of higher protein delivery. However, they are considered relatively weak evidence since illness is considered a confounding factor in the relationship between delivery and outcome for which we cannot completely adjust. Randomized trials have not been conducted, although two trials with randomized high(er) amino acid infusion are available and somewhat contradicting [11, 12]. As with the studies on caloric delivery, the studies on protein have been hampered by insufficient knowledge on energy and protein metabolism under these (patho)physiological circumstances in the ICU patient [7–9].

Therefore, mechanistic studies on the protein physiology in ICU patients is an essential and current development. The Swedish group of Wernerman and Rooyackers has provided crucial information on the topic. They showed that it was possible to change protein balance during the early phase of admission to the ICU from negative to positive by a short-term (3-h) high-level (1 g/kg/day) amino acid (AA) infusion [13]. This observation was very important to help understand the physiology since it showed that, under these circumstances of critical illness, some basic principles of nutrition still perform well.

In the December 2017 issue of *Critical Care*, Sundstrom et al. showed that the effect of supplemental AA infusion at 3 h is still present at 24 h [14]. Why is this so important to know? We know from extensive studies in sports and the elderly that protein synthesis can be stimulated by bolus protein feeding; however, we know relatively little about the effects of continuous (low dose per time unit) feeding. While the absolute levels of protein balance still have to be considered with caution (e.g., choice of tracer), and we are not completely sure where the protein is going, we now know this positive effect on protein balance is lasting.

The next challenge is to reconnect this physiological information with the outcome of ICU patients. We have shown that muscle (protein) mass at admission to the ICU is relevant for the outcome of ICU patients [15]. We do not know if we can change muscle mass and outcome of ICU patients with protein nutrition. The study by Sundstrom et al. [14] is very promising for protein balance, but will that be enough to change outcome? And, if so, is that true for all patients—does one size fit all?

The ICU patient group is heterogeneous. Earlier, we found high protein delivery to be associated with lower mortality, except for sepsis patients and patients with early caloric overfeeding [7]. The EAT-ICU trial did not find an effect of early goal-directed feeding on physical component score at 6 months or on mortality [6]. Goal-directed feeding included feeding energy based on indirect calorimetry and protein up to 1.5 g/kg/day from day 1. Feeding calories up to the measured caloric target from day 1 may be equal to caloric overfeeding [7]. The 47% of patients with sepsis in the EAT-ICU trial might also not benefit from the higher protein feeding [7]. Therefore, the effects of protein and energy cannot be assessed individually from this trial. Ferrie et al. showed interesting differences in muscle mass and function between an AA infusion rate of 0.8 and 1.2 g/kg/day [12], but not all patients are equal—one size does not fit all! Those patients with a low protein reserve (low muscle mass) may be at highest risk in the ICU and may benefit more from intervention with early protein nutrition.

We have to await further studies, including randomized studies and post-hoc observational studies, to further develop this area of interest. The studies trying to understand the mechanism behind the physiological effect are important as well; we might come nearer to the truth of what works and what does not work in ICU nutrition.
